# The Role of Working Memory, Short-Term Memory, Speed of Processing, Education, and Locality in Verb-Related Morphosyntactic Production: Evidence From Greek

**DOI:** 10.3389/fpsyg.2022.851440

**Published:** 2022-07-14

**Authors:** Valantis Fyndanis, Elvira Masoura, Sonia Malefaki, Efpraxia Chatziadamou, Ifigeneia Dosi, David Caplan

**Affiliations:** ^1^Department of Rehabilitation Sciences, Faculty of Health Sciences, Cyprus University of Technology, Limassol, Cyprus; ^2^Center for Multilingualism in Society Across the Lifespan (MultiLing), Department of Linguistics and Scandinavian Studies, Faculty of Humanities, University of Oslo, Oslo, Norway; ^3^Department of Experimental Cognitive Psychology, School of Psychology, Faculty of Philosophy, Aristotle University of Thessaloniki, Thessaloniki, Greece; ^4^Department of Mechanical Engineering and Aeronautical Engineering, School of Engineering, University of Patras, Patras, Greece; ^5^Department of Greek Philology, Democritus University of Thrace, Komotini, Greece; ^6^Department of Neurology, Massachusetts General Hospital, Harvard Medical School, Boston, MA, United States

**Keywords:** morphosyntactic production, grammatical aspect, time reference/tense, subject–verb agreement, working memory, short-term memory, speed of processing, education

## Abstract

This study investigates the relationship between verb-related morphosyntactic production (VRMP) and locality (i.e., critical cue being adjacent to the target or not), verbal Working Memory (vWM), nonverbal/visuospatial WM (nvWM), verbal short-term memory (vSTM), nonverbal/visuospatial STM (nvSTM), speed of processing, and education. Eighty healthy middle-aged and older Greek-speaking participants were administered a sentence completion task tapping into production of subject–verb Agreement, Time Reference/Tense, and grammatical Aspect in local and nonlocal configurations, and cognitive tasks tapping into vSTM, nvSTM, vWM, nvWM, and speed of processing. Aspect elicited worse performance than Time Reference and Agreement, and Time Reference elicited worse performance than Agreement. There were main effects of vSTM, vWM, education, and locality: the greater the participants’ vSTM/vWM capacity, and the higher their educational level, the better their VRMP; nonlocal configurations elicited worse performance on VRMP than local configurations. Moreover, vWM affected Aspect and Time Reference/Tense more than Agreement, and education affected VRMP more in local than in nonlocal configurations. Lastly, locality affected Agreement and Aspect (with nonlocal configurations eliciting more agreement and aspect errors than local configurations) but not Time Reference. That vSTM/vWM (but not nvSTM/nvWM) were found to subserve VRMP suggests that VRMP is predominantly supported by domain-specific, not by domain-general, memory resources. The main effects of vWM and vSTM suggest that both the processing and storage components of WM are relevant to VRMP. That vWM (but not vSTM) interacts with production of Aspect, Time Reference, and Agreement suggests that Aspect and Time Reference are computationally more demanding than Agreement. These findings are consistent with earlier findings that, in individuals with aphasia, vWM interacts with production of Aspect, Time Reference, and Agreement. The differential effect of education on VRMP in local vs. nonlocal configurations could be accounted for by assuming that education is a proxy for an assumed procedural memory system that is sensitive to frequency patterns in language and better supports VRMP in more frequent than in less frequent configurations. In the same vein, the interaction between locality and the three morphosyntactic categories might reflect the statistical distribution of local vs. nonlocal Aspect, Agreement, and Time Reference/Tense in Greek.

## Introduction

In Greek aphasia and Alzheimer’s disease (AD), it has been consistently found that production of subject–verb Agreement, Time Reference/Tense, and grammatical Aspect is selectively impaired, with subject–verb Agreement being better preserved (in most individuals with aphasia or AD) than grammatical Aspect or Time Reference/Tense (for nonfluent aphasia in Greek, see Nanousi et al., 2006; Varlokosta et al., 2006; Fyndanis et al., 2012, 2018; but see Protopapas et al., 2016; for AD in Greek, see Fyndanis et al., 2013; for background information on subject–verb Agreement, Tense/Time Reference, and grammatical Aspect, see next section). Better performance on production of Tense than on production of subject–verb Agreement has also been reported for individuals with aphasia in several other languages (e.g., for Dutch: Kok et al., 2007; for Spanish, Catalian, and Galician: Gavarró and Martínez-Ferreiro, 2007; for German: Wenzlaff and Clahsen, 2004; for Hebrew: Friedmann and Grodzinsky, 1997; for English: Clahsen and Ali, 2009). The dissociation consistently found in the above-mentioned Greek-speaking neurologically affected populations has been accounted for by the Interpretable Features’ Ιmpairment Hypothesis (IFIH; Varlokosta et al., 2006; Fyndanis et al., 2012), which posits that categories bearing interpretable features (e.g., grammatical Aspect and Tense/Time Reference) are computationally more demanding than categories bearing uninterpretable features (e.g., subject–verb Agreement), and therefore, individuals with reduced processing resources and/or reduced Working Memory (WM) capacity (such as people with nonfluent aphasia or individuals with AD) fare better on production of undemanding morphosyntactic categories such as subject–verb Agreement than on production of demanding categories such as Tense/Time Reference and grammatical Aspect. The distinction between interpretable and uninterpretable features has been made in the Minimalist Program (e.g., Chomsky, 1995, 2000, 2001). Put simply, interpretable features contribute to semantic interpretation, whereas uninterpretable features do not (Kok et al., 2007).^1^ Importantly, following Kok et al. (2007) and Fyndanis et al. (2012) took the distinction between interpretable and uninterpretable features as being reflected in ± involvement of integration processes. Categories bearing interpretable features (e.g., Tense and Aspect) require processing and integration of information from two distinct levels of representation (i.e., grammatical level and extralingustic/conceptual level), whereas categories bearing uninterpretable features (e.g., subject–verb Agreement) require processing of grammatical information only. Therefore, the latter categories do not involve integration processes. The predictions made by IFIH (Varlokosta et al., 2006; Fyndanis et al., 2012) rest on the assumption that integration processes are computationally costly (see, e.g., Hartsuiker et al., 1999; Avrutin, 2000; Kok et al., 2007; Yarbay Duman and Bastiaanse, 2009; Bastiaanse et al., 2011).

As mentioned above, the dissociation between grammatical Aspect, Tense/Time Reference, and subject–verb Agreement, consistently found in studies on Greek aphasia and AD, is in line with IFIH. Direct evidence for IFIH, however, was provided by Fyndanis et al.’s (2018) finding that, both in a group consisting of eight Greek-speaking individuals with aphasia and eight healthy controls and in a group of 103 Greek-speaking neurologically healthy participants sampling the whole adult age range, the grammatical Aspect and Time Reference conditions elicited more errors than the subject–verb Agreement condition, and, importantly, verbal WM affected grammatical Aspect and Tense/Time Reference significantly more than subject–verb Agreement. This interaction is consistent with the notion that morphosyntactic categories involving integration processes (e.g., grammatical Aspect and Tense) are more costly than categories that do not involve integration processes (e.g., subject–verb Agreement). This between-category difference in processing demands is also reflected in the acquisition order of categories bearing interpretable vs. uninterpretable features. Morphosyntactic phenomena bearing uninterpretable features, that is, phenomena that belong to core syntax and are semantically vacuous (e.g., number and person Agreement), are acquired earliest (at the age of three for Greek monolingual children; Doukas and Marinis, 2012). In contrast, morphosyntactic phenomena bearing interpretable features (e.g., Tense, Aspect) are acquired later. It should be noted that, in Fyndanis et al.’s (2018) study, verbal WM capacity also affected grammatical Aspect more than Time Reference/Tense, and, in both participants with aphasia and neurotypical participants, the Aspect condition elicited significantly more errors than the Time Reference/Tense condition. Therefore, not only were grammatical Aspect and Time Reference/Tense more demanding than subject–verb Agreement, but grammatical Aspect was also more demanding than Time Reference/Tense. Fyndanis et al.’s (2018) results, therefore, suggest that the three morphosyntactic categories above do not deploy the same amount of processing resources, and verbal WM subserves verb-related morphosyntactic production, shaping the observed patterns of performance.

There are several reasons why grammatical Aspect is more demanding than Time Reference/Tense, at least in Greek. Although both Tense and Aspect bear interpretable features, they differ in acquisition order, as Tense is acquired before Aspect in Greek (Delidaki and Varlokosta, 2003). Specifically, the acquisition of Tense in Greek is almost acquired at the age of 3.2 years (Delidaki and Varlokosta, 2003). The acquisition of Aspect is more demanding, and thus, Aspect belongs to the late acquisition phenomena. This is so because it involves not only semantics but also pragmatics, which renders it computationally demanding (Tsimpli, 2014). Some studies on Greek-speaking children have observed that Aspect is fully acquired by the age of 6 (Kaltsa, 2012; Kotroni, 2014), while other studies found that even 10-year-old Greek monolingual children encounter difficulties in the correct use of aspectual feature when prepositional phrases (e.g., *for/in X time*) are included in sentence repetition and correction tasks (Dosi, 2016; Unpublished PhD Thesis^2^; Dosi et al., 2016). It was also found that acquisition of Aspect in monolingual children (aged 8–12 years) is predicted by their WM abilities (see footnote 1). Furthermore, unlike Tense, Aspect is more “subjective” than Tense (e.g., Comrie, 1976; Smith, 1997), involving thus intentional/conceptual representations rather than “objective” extra-linguistic representations, as is the case with Tense (see Fyndanis et al., 2012). It could be that “involvement of intentional/conceptual representations poses more demands on the processing system compared to involvement of extensional/extralinguistic information” (Fyndanis et al., 2012, p. 1144).

Lastly, it should be noted that Fyndanis et al. (2018) also investigated the role of locality, that is, the feature of the stimulus design that is related to whether the critical cue (e.g., time adverbial in the Time Reference/Tense condition) is adjacent to the to-be-produced target verb form (for examples of local and nonlocal configurations, see Table 1). They found no significant main effect of locality in either group (i.e., participants with aphasia and neurotypical participants), and no significant interactions between locality and verbal WM or between locality and the three morphosyntactic categories above.

**Table 1:** Examples of experimental items tapping into production of subject–verb Agreement, Time Reference, and grammatical Aspect.

Morphosyntactic condition	Source sentence	Target sentence
*Local Agreement*	*‘Avrio mésa se misí óra esí θa mirásis ta ðóra*	*‘Avrio mésa se misí óra aftós _________________.* (target: θa mirási ta ðóra)
	Tomorrow within half an hour you-sg will distribute-2^nd^.sg the gifts (lit.)	Tomorrow within half an hour he ____________. (target: will distribute-3^rd^.sg the gifts) (lit.)
*Nonlocal Agreement*	*Esí ávrio mésa se misí óra θa mirásis ta ðóra*	*Aftós ávrio mésa se misí óra _________________.* (target: θa mirási ta ðóra)
	You-sg tomorrow within half an hour will distribute-2^nd^.sg the gifts (lit.)	He tomorrow within half an hour ____________. (target: will distribute-3^rd^.sg the gifts) (lit.)
*Local Time Reference*	*Mésa se misí óra aftós xθés mírase ta ðóra* Within half an hour he yesterday distributed the gifts (lit.)	*Mésa se misí óra aftós ávrio _____________________.* (target: θa mirási ta ðóra) Within half an hour he tomorrow ___________. (target: will distribute the gifts) (lit.)
*Nonlocal Time Reference*	*Χθés aftós mésa se misí óra mírase ta ðóra* Yesterday he within half an hour distributed the gifts (lit.)	*‘Avrio aftós mésa se misí óra _____________________.* (target: θa mirási ta ðóra) Tomorrow he within half an hour _______________. (target: will distribute the gifts) (lit.)
*Local Aspect*	*Aftós ávrio mésa se misí óra θa mirási ta ðóra* He tomorrow within half an hour will distribute-perf the gifts (lit.)	*Aftós ávrio epí misí óra ________________________.* (target: θa mirázi ta ðóra) He tomorrow for half an hour _________________ (target: will distribute-imperf the gifts) (lit.)
*Nonlocal Aspect*	*Mésa se misí óra aftós ávrio θa mirási ta ðóra* Within half an hour he tomorrow will distribute-perf the gifts (lit.)	*Epí misí óra aftós ávrio _______________________.* (target: θa mirázi ta ðóra) For half an hour he tomorrow _________________ (target: will distribute-imperf the gifts) (lit.)

This table is largely based on Fyndanis et al. (2020, p. 5).

### Background on WM and Education, and Their Role in Verb-Related Morphosyntactic Production

Τhere is no consensus on the definition and conceptualization of WM (Cowan, 2017), while the key differences in theoretical assumptions and approaches to WM are many (Logie et al., 2020). According to the most influential approach to WM, this memory system consists of storage and processing components, and there are separate components for storing and maintaining verbal vs. nonverbal/visuospatial material (e.g., Baddeley and Hitch, 1974; Baddeley, 1986, 1992; Baddeley et al., 2020). This is the “multicomponent view of WM.” Consistent with the multicomponent view of WM is also Martin and colleagues’ (Martin and Romani, 1994; Martin et al., 1994, 1999) sophisticated model of verbal WM, which postulates an input phonological buffer, an output phonological buffer, and a lexical-semantic buffer. An alternative view of WM is provided by the experience-based (or “emergent”) approach to verbal WM. This approach posits that verbal WM is the skill of maintaining and ordering linguistic/verbal material, and that skill (just like all subcomponents of language production and comprehension) emerges from “actions” of the language systems and varies with experience (e.g., MacDonald and Christiansen, 2002; MacDonald, 2016; Schwering and MacDonald, 2020).

In the current study, we adopt both the widely accepted view that WM consists of storage and processing components (e.g., Baddeley, 1986; Cowan, 2008), and that short-term memory (STM) is the storage component of WM (e.g., Baddeley and Hitch, 1974; Baddeley, 1992), and the multicomponent view of WM, according to which there two or more storage components (e.g., Baddeley and Hitch, 1974; Baddeley, 1986, 1992; Martin and Romani, 1994; Martin et al., 1994, 1999; Baddeley et al., 2020). The alternative view of WM, i.e., the experienced-based/emergent view of WM (e.g., MacDonald and Christiansen, 2002; MacDonald, 2016; Schwering and MacDonald, 2020), will also be considered.

Evidence that verbal WM is critically involved in verb-related morphosyntactic production has been provided both in studies investigating cue-based retrieval interference^3^ (e.g., Hartsuiker and Barkhuysen, 2006; Slevc and Martin, 2016) and in studies that focused on structures that do not involve or favor cue-based retrieval interference (e.g., Kok et al., 2007; Fyndanis et al., 2018). Little is known, however, about the role of other related cognitive capacities in verb-related morphosyntactic production.^4^ Speed of processing (SOP), for example, has been found to be closely related to WM (e.g., Salthouse, 1992; Fry and Hale, 1996, 2000). Likewise, STM is closely related to WM, as it constitutes its simple storage component (e.g., Baddeley and Hitch, 1974; Baddeley, 1992); the related question of whether both the storage and processing components of WM affect verb-related morphosyntactic production was not addressed. Relatedly, it is not yet clear whether it is domain-specific (i.e., verbal) memory resources or domain-general memory/attentional resources (or both) that support verb-related morphosyntactic production. There is empirical evidence suggesting that (1) the verbal and nonverbal STM systems predominantly rely on domain-specific resources (e.g., Kane et al., 2004; Hanley and Young, 2019; Logie, 2019), whereas the verbal and nonverbal WM systems predominantly rely on domain-general resources (e.g., Kane et al., 2004), and (2) nonverbal WM relies on domain-general resources to a greater extent than verbal WM (e.g., Vergauwe et al., 2010).

Verb-related morphosyntactic production might pose demands on both the storage and processing components of verbal WM, that is, on both verbal STM and verbal WM. Specifically, in sentence completion tasks like that used in Fyndanis et al. (2018) and in the current study (see Materials and Methods section and Table 1), participants have to *store* in their verbal STM/WM the lemma representation of the verb that appears in the source sentence, as well as the values of the morphosyntactic features encoded in the material that precedes the target verb form in the target sentence (e.g., +Perfective for grammatical Aspect, +Past for Time Reference, and +Singular and +3^rd^ person for subject–verb Agreement).^5^ However, to encode these values, their decoding/extraction from the phonological forms/lexemes preceding the target verb form is required. Moreover, to produce the target verb form, participants have to combine the activated lemma representation of the verb included in the source sentence with the values of the morphosyntactic features encoded in the material preceding the target verb form in the target sentence, and subsequently to select/retrieve a lexeme that corresponds to the activated (and selected) lemma representation. That is, this lexeme should encode the target morphosyntactic feature(s). Probably the latter “steps” in the task completion are computationally demanding, posing thus processing demands on participants’ WM system. One could assume, thus, that verb-related morphosyntactic production involves both storage and processing WM resources. Moreover, one could assume that production of demanding morphosyntactic categories such as grammatical Aspect poses more demands on verbal STM/WM in nonlocal than in local configurations because nonlocal configurations require the participant to maintain the critical feature for a longer time across intervening words and features.

As mentioned above, an alternative view of WM is provided by the experience-based or emergent approach to verbal WM, which posits that verbal WM is the skill of maintaining and ordering linguistic/verbal material, and that skill emerges from “actions” of the language systems and varies with “linguistic experience” (e.g., MacDonald and Christiansen, 2002; MacDonald, 2016; Schwering and MacDonald, 2020). Hence, the experienced-based approach interprets performance on verbal STM/WM tasks as reflecting degree of language skill, and in particular as assessing the quantity and quality of a person’s language skill and experience that is relevant to the demands of the task (Schwering and MacDonald, 2020). Therefore, as per this approach, verbal STM/WM tasks do not measure a person’s capacity of a separate verbal STM/WM system but rather their skill in encoding and maintaining verbal material, which is shaped by various forms of linguistic knowledge, including knowledge of lexemes and word meanings (Schwering and MacDonald, 2020). Furthermore, the proponents of “emergent” WM models do not distinguish between the processing component and the storage component of WM, which is based on the idea that storage of information in WM always requires its transformation in some way in the service of goal-directed behavior (Buchsbaum and D’Esposito, 2019). Moreover, unlike the proponents of multicomponent WM models, who argue that WM/STM is distinct from long-term memory, the proponents of emergent WM models argue that verbal WM is the activated portion of “linguistic long-term memory” (e.g., Cowan, 1993; Acheson and MacDonald, 2009a,b; Hasson et al., 2015; MacDonald, 2016). Since emergent WM models assume that verbal STM/WM and morphosyntactic production largely rely on the same verbal/linguistic resources, they should expect *verbal* STM/WM (but not nonverbal STM/WM) to subserve verb-related morphosyntactic production. Moreover, since emergent WM models do not distinguish between the storage and processing components of WM, they should expect performance on both verbal STM and verbal WM tasks to predict performance on tasks tapping into verb-related morphosyntactic production.

Finally, education might also play a role in verb-related morphosyntactic production. As pointed out by Simos et al. (2011), (years of formal) education “may directly affect performance on verbal tests […], as a proxy for […] formal linguistic experience, and cognitive reserve, and also as an indicator of increased experience in formal testing situations (Ostrosky-Solis et al., 1998, p. 487) […].” Relatedly, a higher educational level produces more exposure to written language and possibly a greater metalinguistic knowledge (depending on the kind of studies one pursues). Moreover, individuals with a higher educational level may tend to read more and to engage in conversations about a wider range of topics later on in their life compared to people with a lower educational level. Therefore, education is likely to determine the degree of linguistic experience, which in turn determines the strength of the connections in the linguistic network hosted in long-term memory. Education, thus, could be taken as another proxy for language skill and experience, which determines the efficiency or capacity of verbal STM/WM as defined by MacDonald and colleagues (MacDonald and Christiansen, 2002; Acheson and MacDonald, 2009a,b; MacDonald, 2016; Schwering and MacDonald, 2020).

Alternatively, education might be considered to be a proxy for *long-term WM for language* (Caplan and Waters, 2013). The term *long-term WM* has been coined by Ericsson and Kintsch (1995), who argued that this memory system is a WM system that is based on storage in long-term memory and is connected to skilled activities. Based on findings from studies on the relationship between WM and online sentence/syntactic comprehension, Caplan and colleagues (see Caplan and Waters, 2013, and references therein) proposed that language constitutes a skilled activity, and on-line syntactic processing in comprehension is predominantly supported by long-term WM for language, which is a procedural memory system. They also suggested that STM/WM only supports memory demands of processes occurring at points of incremental comprehension failure. However, several properties/features of long-term WM system for language (including the empirical markers of incremental comprehension failure) have yet to be determined.

Although these ideas were developed to capture language comprehension data, one cannot rule out that long-term WM for language is also involved in aspects of language production, such as verb-related morphosyntactic production. It might be, for example, that production of verb-related morphosyntactic categories is subserved by both controlled and procedural memory systems.

It should be noted that, although both Caplan and Waters (2013) and MacDonald and colleagues (e.g., MacDonald and Christiansen, 2002; Acheson and MacDonald, 2009a,b; MacDonald, 2016; Schwering and MacDonald, 2020) postulate an experience-based, skill-related and domain-specific (i.e., verbal/linguistic) system that supports language, their approaches differ in at least two aspects. Firstly, MacDonald and colleagues argue that both language processing (in production and comprehension) and performance on verbal WM/STM tasks rely on the same experience-based system, whereas Caplan and Waters (2013) assume that language processing is supported by at least two distinct memory systems: an experienced-based procedural memory system (i.e., long-term WM for language) and a STM/WM system which is distinct from long-term memory. Secondly, while both education and performance on verbal STM/WM tasks could be taken as proxies for language skill and experience in MacDonald and colleagues’ approach, only education could be taken as a proxy for language skill and experience—which in turn determines the efficiency of the long-term WM system for language—in Caplan and Waters’ (2013) approach.

### The Current Study

The current study follows up on Fyndanis et al.’s (2018) study and investigates the relationship between verb-related morphosyntactic production and verbal WM, nonverbal/visuospatial WM, verbal STM, nonverbal/visuospatial STM, SOP, and education, with the latter being taken as a proxy for long-term WM for language. In particular, we investigate the role of these cognitive capacities and education in the production of grammatical Aspect, Time Reference/Tense, and subject–verb Agreement in Greek-speaking healthy aging individuals. A research question that is related to this investigation is whether both the storage and processing components of WM are involved in verb-related morphosyntactic production, and—relatedly—whether the latter is supported by both domain-specific and domain-general memory mechanisms. Another question is whether verb-related morphosyntactic production is supported by both STM/WM and an assumed “long-term WM for language” (Caplan and Waters, 2013). Finally, the study addresses whether locality (i.e., the design feature concerning the position of the critical cue relative to the target) is related to any of the memory systems examined here (including long-term WM for language); and, if so, whether such relationship is modulated by the morphosyntactic categories examined here.

### Background Information on Time Reference/Tense, Grammatical Aspect, and Subject–Verb Agreement

Tense is a morphosyntactic (“functional”) category that is used to locate events in time. Present tense, for example, usually locates an event as simultaneous with the utterance time (a.k.a. speaking time); past tense locates it prior to the utterance time (that is, in a past time frame); and future tense locates it subsequently to the utterance time (that is, in a future time frame; e.g., Comrie, 1985). Time Reference is a semantic category that is closely related—but not identical—to Tense. This is so because in many languages (including Greek and English) Time Reference is made through tenses. However, Tense and Time Reference do not fully overlap as different tenses can be used to refer to the same time frame. Present perfect and simple past tense, for example, both refer to the past. Furthermore, the same tense can be used to refer to more than one time frame. For instance, present tense in Italian (e.g., *bevo* “drink”) can refer to either the present [e.g., *Adesso bevo una birra* “Now drink-present.1st.sg a beer” (lit.)] or to the future [e.g., *Stasera bevo una birra* “Tonight drink-present.1st.sg a beer” (lit.)].

Grammatical Aspect relates to *how* the speaker views an event (e.g., Comrie, 1976; Smith, 1997). For instance, the sentences *Yesterday Mary was reading a novel when John gave her a ring* and *Yesterday Mary read a novel* differ in Aspect, that is, in the way the speaker views the reading event. In the former sentence, (s)he views the reading event as *progressive*, whereas in the latter sentence (s)he views it as *non-progressive*. This aspectual difference reflects the fundamental distinction between *imperfectivity* and *perfectivity*, respectively. The perfective Aspect is used when the speaker views an event as a whole, and the imperfective Aspect is used when the speaker focuses on the internal structure of an event, that is, on the separate phases making up that event. The sentences *Yesterday Mary was reading a novel when John gave her a ring* and *Yesterday Mary read a novel* illustrate that the way of viewing an event depends on the speaker’s point of view. This is the reason why, unlike subject–verb Agreement and Time Reference/Tense, grammatical Aspect is a “subjective” category (e.g., Comrie, 1976; Smith, 1997).

Lastly, in languages such as Greek, the person and number features of the grammatical subject of a sentence are morphologically encoded/marked on the verb. This morphosyntactic phenomenon is called *subject–verb Agreement*. The combination of two numbers (singular and plural number) and three persons (first, second, and third person) is encoded on all Greek finite verb forms, in all tenses. All Greek finite verb forms also encode (morphologically) Tense/Time Reference and grammatical Aspect. However, in Greek, the perfective–imperfective aspectual distinction is only encoded in past-tensed and future-tensed verb forms in indicative mood (as well as in the tenseless subjunctive mood which is not relevant here; Holton et al., 1997). In Greek regular verb forms, perfective Aspect is encoded by the aspectual marker -*s*-, which is attached to the imperfective verb stem (Ralli, 1988; Holton et al., 1997; note that only the imperfective verb stem is used in the present Tense in Greek, whereas in past- and future-referring verb forms both the perfective and imperfective stems can be used). In regular verb forms in Greek, in the presence of the aspectual marker -*s*- the stem-final consonant is deleted (e.g., *lín-o* “I untie” > *éli-**s**-a* “I untied”) or is phonologically altered (e.g., *ráv-o* “I stitch” > *é-rap-**s**-a* “I stitched”; Ralli, 1988). The interaction between Tense/Time Reference and grammatical Aspect in Greek (for the indicative mood) is illustrated in Table 2.

**Table 2:** Interaction between grammatical Aspect and Time Reference/Tense in Greek (for the verb *rávo*“stitch” with the imperfective stem *ráv*- and the perfective stem *ráp*-).

	Imperfective	Perfective
Present	*ráv-o* “I stitch”	–
Past	*é-rav -a* “I stiched-ΙΜPERFECTIVE”	*é-rap-s-a* “I played-PERFECTIVE”
Future	*θa ráv-o* “I will stitch-ΙΜPERFECTIVE”	θa r*áp-s-o* “I will stitch-PERFECTIVE”

## Materials and Methods

### Participants

Eighty neurotypical adults (55 women, 25 men) participated in the study (*Mean Age* = 60.1 years; *SD* = 4.2 years; *Range* = 55–67 years). They were selected in a way so as to vary in the number of years of formal education (*Mean* = 13.1; *SD* = 4.53, *Range* = 6–22 years). Before testing, all participants were administered a validated Greek version of the Mini-Mental State Examination (Folstein et al., 1975; Greek version: Tsolaki et al., 1997) to exclude individuals with cognitive impairments. All participants scored ≥28.

The study was approved by the Research Ethics Committee of the School of Psychology (Faculty of Philosophy) at the Aristotle University of Thessaloniki.

Participants were recruited through the social, the work and the family environment of the authors and were informed about the scope, the duration, and the procedure of the research. They all signed a consent form before participation, and all APA ethical guidelines were followed throughout the research procedure.

### Materials

Participants completed the sentence completion task reported in Fyndanis et al. (2018), and cognitive tasks tapping into verbal STM (Digit Recall/Digit Forward Span task) nonverbal/visuospatial STM (Block Recall task), verbal WM (Backwards Digit Recall/Digit Backward Span task), nonverbal/visuospatial WM (Backwards Block Recall task), and SOP (Greek version of the Digit-Symbol subtest from the Wechsler Adult Intelligence Scale-Fourth Edition; Stogiannidou, 2012; Wechsler, 2014).

#### Sentence Completion Task

The sentence completion task, which was developed by Fyndanis et al. (2018), included 192 experimental items and tested participants’ ability to produce three verb-related morphosyntactic categories: grammatical Aspect, Time Reference/Tense, and subject–verb Agreement. Each condition consisted of 64 items. In the Aspect condition, there were 32 items eliciting perfective Aspect and 32 items eliciting imperfective Aspect. In the Time Reference condition, there were 32 items eliciting past-referring verb forms and 32 items eliciting future-referring verb forms. The Agreement condition consisted of 32 items tapping into person agreement and 32 items tapping into number agreement. The three morphosyntactic conditions were matched on sentence length. Overall, 16 bisyllabic, regular, two-place transitive (i.e., taking only one object) verbs were used. All verbs were stressed on the penultimate syllable. Overall, each verb appeared 12 times, four times in each morphosyntactic condition: twice in local configurations and twice in nonlocal configurations (for example, in the Tense condition, the same verb appeared once in “local past”, once in “nonlocal past”, once in “local future”, and once in “nonlocal future”; for some examples, see Table 1). The task was split in two lists, with each list comprising 96 items (32 items for each morphosyntactic condition). Half participants completed List 1 and half List 2. All 16 verbs appeared in both lists, with each verb appearing six times (twice in each morphosyntactic condition) in either list. The three morphosyntactic conditions were pseudorandomized in each list, and the item order was kept constant across participants. Participants listened to a source sentence [e.g., *Mésa se misí óra aftós xθés mírase ta ðóra* “Within half an hour he yesterday distributed the gifts (lit.)”] and the beginning of a target sentence [e.g., *Mésa se misí óra aftós ávrio* “Within half an hour he tomorrow (lit.)”], and were instructed to complete the target sentence by orally providing the missing verb phrase (i.e., *θa mirási ta ðóra* “will distribute the gifts”). They always had to transform the verb form that appeared in the source sentence into a different verb form. Examples of the Agreement, Time Reference, and Aspect conditions are provided in Table 1. Accuracy was the dependent variable, and scoring was based on the verb forms the participants produced.

As one can infer from Table 1, the same verb forms could be the correct answers in different conditions. However, what is important in each condition is the *transition* from the source sentence to the target sentence, which requires *transformation* of the verb form that appears in the source sentence, that is, retrieval of a verb form other than that appearing in the source sentence. Crucially, although all experimental items included three phrases that preceded the target verb form, namely grammatical subject, time/temporal adverbial and aspectual adverbial, only one of these phrases served the function of the critical cue in each condition. That is, each condition involved a different type of critical cue. In the subject–verb Agreement condition, the critical cue was the grammatical subject (e.g., *you*, *he*, *they*), and the source sentence differed from the target sentence as for this cue only. For instance, in the example of the Agreement condition included in Table 1, there is a transition from *esí* “you-singular” (in the source sentence) to *aftós* “*he*” (in the target sentence). In the Time Reference condition, a time/temporal adverbial (e.g., *yesterday*, *tomorrow*) was the critical cue, and the source sentence differed from the target sentence as for this cue only. In the example of the Time Reference/Tense condition included in Table 1, there is a transition from *xθés* “yesterday” (in the source sentence) to *ávrio* “*tomorrow*” (in the target sentence). Lastly, in the grammatical Aspect condition, the critical cue was an aspectual adverbial (e.g., *within 5 min*, *for 5 min*). The example of the Aspect condition included in Table 1 features a transition from *mésa se misí óra* “within half an hour” (in the source sentence) to *epí misí óra* “for half an hour” (in the target sentence). Note that, in Greek, the aspectual adverbial *mésa se misí óra* “within half an hour” is consistent with the perfective aspect, whereas the adverbial *epí misí óra* “for half an hour” is consistent with the imperfective aspect.

It should be noted that irrelevant errors were not considered. For instance, in the Time Reference condition, Aspect and Agreement errors were ignored. This means that, in the example of the Time Reference condition (in Table 1), not only the target verb form *θa mirási* “will distribute-3^rd^.sg-perfective aspect,” but also the verb forms *θa mirázi* “will distribute-3^rd^.sg-imperfective aspect” and *θa mirásis* “will distribute-2^nd^.sg-perfective aspect” (among other verb forms) should be scored as correct, despite the fact that the last two verb forms are correct as for Time Reference but incorrect as for Aspect or Agreement. Following the same scoring criterion, in the example of the Agreement condition (in Table 1), not only the target verb form *θa mirási* “will distribute-3^rd^.sg-perfective aspect,” but also the verb forms *mírase* “distributed-3^rd^.sg-perfective aspect,” *míraze* “distributed-3^rd^.sg-imperfective aspect” and *θa mirázi* “will distribute-3^rd^.sg-imperfective aspect” (among others) should be scored as correct, although the last three verb forms are correct as for Agreement but erroneous as for Time Reference and/or Aspect. The same logic applied to the Aspect condition. Table 1, therefore, does not include an exhaustive list of the verb forms that could be scored as correct in the examples given. For brevity, this table only includes the verb forms the production of which would result in fully grammatical sentences (i.e., the verb forms that agree with all three preverbal phrases—i.e., grammatical subject, time/temporal adverbial and aspectual adverbial).

#### Verbal STM and Verbal WM

To measure participants’ verbal STM capacity and verbal WM capacity, we used Greek versions of the Digit Recall task (a.k.a. forward digit span task) and the Backwards Digit Recall task (a.k.a. backwards digit span task), respectively. These tasks were modeled and developed after the Digit Recall and Backwards Digit Recall tasks included in Pickering and Gathercole’s (2001) battery, which has been reported to have a high degree of internal and external validity (Gathercole and Pickering, 2000; Masoura et al., 2009).

In the Digit Recall task, participants were auditorily presented with sequences of random digits (e.g., 5, 9, 2, 6) and were asked to repeat them back in the same order of presentation (i.e., 5, 9, 2, 6). Digits were presented at a rate of one per second. The first trial consisted of two digits and level of difficulty increased up to 13 digits. Thus, there were 12 difficulty levels. Each level consisted of six test trials. Each correct answer was given one point, and there were no penalty points for wrong answers. The maximum score, therefore, was 72 and the minimum score was 0.

In the Backwards Digits Recall task, the participant heard a sequence of random digits (e.g., 2, 8, 1, 4) and was instructed to repeat them back in reverse order of presentation (i.e., 4, 1, 8, 2). Again, digits were presented at a rate of one per second. The first difficulty level contained two digits and the last difficulty level consisted of nine digits. Thus, there were eight difficulty levels. Each level consisted of six test trials. Again, each correct answer was given one point, and there were no penalty points for wrong answers. Therefore, the maximum score was 48, and the minimum score was 0.

In both tasks, we used a random number generator to develop the digit series. The administration instructions used were the same as those included in the Manual of the Working Memory Test Battery for Children (Pickering and Gathercole, 2001).

#### Nonverbal STM and Nonverbal WM

To measure participants’ nonverbal/visuospatial STM capacity and nonverbal/visuospatial WM capacity, we used Greek versions of the Block Recall task and a Backwards Block Recall task, respectively. They were both based on the classical Corsi span (Corsi, 1972), and were modeled after the Block Recall and Backwards Block Recall tasks included in Pickering and Gathercole (2001).

In the Block Recall task, participants were presented with sequences of blocks on a board and were instructed to recall them in the same order of presentation. In the first trial, participants had to recall the locations of two blocks on the board, in the second trial they had to recall the location of three blocks, and so on. The level of difficulty increased up to nine locations in the last trial. Therefore, there were eight difficulty levels. Each level consisted of six test trials. Each correct answer was given one point, and there were no penalty points for wrong answers. The maximum score was 48, and the minimum score was 0.

In the Backwards Block Recall task, participants were presented with sequences of blocks on a board and were required to recall them in reverse order of presentation. In the first trial, participants had to recall the locations of two blocks on the board. The level of difficulty increased up to nine locations in the last trial. Thus, there were eight difficulty levels. Each level consisted of six test trials. Each correct answer was given one point, and there were no penalty points for wrong answers. The maximum score was 48 and the minimum score was 0.

#### Speed of Processing

To estimate participants’ SOP, we used a Greek standardized version of the Digit-Symbol substitution subtest from the Wechsler Adult Intelligence Scale-Fourth Edition (WAIS–IV; Drozdick et al., 2018; Greek version: Stogiannidou, 2011, 2012; Wechsler, 2014). This task consists of a key that includes nine number-symbol pairs and eight different sequences of randomly ordered numbers, which are below the key. Only numbers 1–9 are included in this task. All symbols are easy to draw. Participant were required to visually scan the key and then write down below each number the corresponding symbol as correctly and as fast as possible. Total score was the number of correct answers within 120 s, and maximum correct score was 135.

### Data Analysis

The dataset was analyzed by fitting generalized linear mixed-effects models (Pinheiro and Bates, 2000). We used the lme4 package in R (Bates et al., 2014) to fit generalized linear mixed-effects models, the lmerTest R package (Kuznetsova et al., 2017) to obtain value of *p* for model parameters, the sjPlot package in R (Lüdecke, 2021) to visualize results, and the emmeans R package (Russell, 2020) to make *post hoc* comparisons.

Because accuracy is a dichotomous variable (1 = correct answer, 0 = wrong answer), logistic models were employed to model correct answers’ probability (Jaeger, 2008). The initial variables were standardized and then entered the models for a better interpretation of the interaction terms and also to retain homogeneity of their measurement scale.

## Results

Descriptive statistics for participants’ education level and performance on morphosyntactic production and cognitive tasks are presented in Table 3. Correlations between the variables of interest are given in Table 4.

**Table 3:** Descriptive statistics for language and cognitive measures, and education.

	*Mean (SD)*	*Min*	*Max*
Agreement total	98.7% (2.8%)	81.3%	100%
Local Agreement	99.2% (273%)	81.2%	100%
Nonlocal Agreement	98.1% (39%)	81.2%	100%
Tense total	95.2% (10.4%)	50.0%	100%
Local Tense	94.3% (15.3%)	12.5%	100%
Nonlocal Tense	96.2% (7.3%)	62.5%	100%
Aspect total	76.5% (20.4%)	21.9%	100%
Local Aspect	78.3% (23.4%)	6.2%	100%
Nonlocal Aspect	74.8% (20.0%)	18.8%	100%
vWM	20.6 (8.1)	7	38
vSTM	36 (6.6)	18	50
nvWM	18.7 (5.4)	7	36
nvSTM	27.4 (4.5)	18	42
SOP	56.3 (14.6)	20	89
EDU	13.1 (4.5)	6	22

SD, standard deviation; vWM, verbal Working Memory; vSTM, verbal Short-Term Memory; nvWM, nonverbal Working Memory; nvSTM, nonverbal Short-Term Memory; SOP, speed of processing; and EDU, (years of formal) education.

**Table 4:** Correlation matrix (*r*s).

	vWM	vSTM	nvWM	nvSTM	SOP	EDU
Agreement	0.12	0.17	0.10	0.14	0.31	0.32
Local Agreement	0.07	0.09	0.03	0.05	0.27	0.22
Nonlocal Agreement	0.13	0.18	0.13	0.17	0.27	0.31
Tense	0.32	0.34	0.26	0.22	0.53	0.40
Local Tense	0.28	0.29	0.23	0.19	0.49	0.38
Nonlocal Tense	0.32	0.35	0.24	0.21	0.47	0.34
Aspect	0.63	0.46	0.27	0.16	0.57	0.69
Local Aspect	0.54	0.40	0.28	0.21	0.55	0.63
Nonlocal Aspect	0.65	0.48	0.21	0.07	0.50	0.68
Morphosynt. Prod. Total	0.56	0.45	0.28	0.20	0.60	0.64
Morphosynt. Prod. Local	0.48	0.39	0.28	0.23	0.59	0.59
Morphosynt. Prod. Nonlocal	0.60	0.48	0.24	0.14	0.54	0.65
vWM		0.47	0.29	0.23	0.53	0.59
vSTM			0.33	0.31	0.48	0.35
nvWM				0.51	0.51	0.23
nvSTM					0.29	0.04
SOP						0.52

Morphosynt. Prod., morphosyntactic production; vWM, verbal Working Memory; vSTM, verbal Short-Term Memory; nvWM, nonverbal Working Memory; nvSTM, nonverbal Short-Term Memory; SOP, speed of processing; and EDU, (years of formal) education.

To address whether locality is related to any of the five memory systems examined here (including long-term WM for language, for which years of formal education were used as a proxy), we first fitted a model including five two-way interactions (i.e., Locality x Verbal WM capacity, Locality x Nonverbal WM capacity, Locality x Verbal STM capacity, Locality x Nonverbal STM capacity, and Locality x Education) as fixed terms, and subjects and items as random intercepts. Subsequently, we fitted the same model with the addition of Locality as by-subject random slope. We selected the best-fitting model based on the Akaike information criterion (AIC; see Burnham and Anderson, 2004; Model 1; see Table 5) and calculated the variance inflation factor (VIF) for all the predictors included in Model 1. The VIF values, which are computed to detect harmful multicollinearity in regression analyses, ranged from 1.06 to 4.31, which are considered acceptable (e.g., Akinwande et al., 2015). Therefore, we did not have to remove any of the variables above.

**Table 5:** Generalized linear mixed-effects Model 1 and Model 2 on accuracy.

	Estimate	Std. error	*z* value	*Pr* (> | *z* |)
*Model 1 (fitted to original dataset)*				
Intercept (Locality = Local)	4.143	0.278	14.894	<0.001^*^
Locality = Nonlocal	−0.593	0.332	−1.786	0.074
Verbal WM capacity	0.401	0.207	1.937	0.053
Verbal STM capacity	0.231	0.193	1.197	0.231
Nonverbal WM capacity	0.014	0.190	0.074	0.941
Nonverbal STM capacity	0.196	0.190	1.030	0.303
Education (years)	0.850	0.195	4.355	<0.001^*^
Locality = Nonlocal: Verbal WM capacity	−0.037	0.173	−0.213	0.831
Locality = Nonlocal: Verbal STM capacity	0.082	0.164	0.501	0.617
Locality = Nonlocal: Nonverbal WM capacity	−0.063	0.156	−0.407	0.684
Locality = Nonlocal: Nonverbal STM capacity	−0.246	0.158	−1.562	0.118
Locality = Nonlocal: Education	−0.350	0.159	−2.201	0.028^*^
*Model 2 (fitted to expanded dataset)*				
Intercept (Locality = Local)	4.485	0.260	17.228	<0.001^*^
Locality = Nonlocal	−0.709	0.303	−2.338	0.019^*^
Education (years)	1.341	0.196	6.853	<0.001^*^
Verbal WM capacity	0.314	0.189	1.664	0.096
Locality = Nonlocal: Education	−0.306	0.118	−2.602	<0.01^*^
Locality = Nonlocal: Verbal WM capacity	0.030	0.113	0.266	0.790

Model 1 (fitted to the original dataset of the current study) included the two-way interactions between (1) Locality (two levels: Local, Nonlocal) and (years of formal) Education (continuous variable), (2) Locality and Verbal WM Capacity (continuous variable), (3) Locality and Nonverbal WM Capacity (continuous variable), (4) Locality and Verbal STM Capacity (continuous variable), and (5) Locality and Nonverbal STM Capacity (continuous variable) as fixed terms, Subjects and Items as random intercepts, and Locality as by-subject random slope. Model 2 (fitted to the expanded dataset) included the two-way interactions between (1) Locality and Education, and (2) Locality and Verbal WM Capacity as fixed terms, Subjects and Items as random intercepts, and Locality as by-subject random slope. The symbol * indicates significant effects.

As shown in Table 5, Model 1 yielded only one significant two-way interaction term, that is, Locality x Education: (number of years of formal) education affected participants’ accuracy performance on verb-related morphosyntactic production more in local configurations than in nonlocal configurations (for examples, see Table 1). Since the content of this interaction seems to be counter-intuitive and given that the sentence completion task used here had also been administered to 103 healthy participants in an earlier study (Fyndanis et al., 2018), we wanted to find out if this interaction remains significant when statistical power increases. To this end, we added to the current dataset (*n* = 80) the data of all healthy participants older than 45 years included in Fyndanis et al.’s (2018) study (*n* = 60), and fitted a model including the two-way interactions between Locality and Education and between Locality and Verbal WM capacity as fixed terms, as well subjects and items as random intercepts and Locality as by subject random slope (Model 2; see Table 5). Note that Fyndanis et al. (2018) only tested their participants on two WM tasks: a digit ordering span task and a digit backward span task/backwards digits recall task. To maximize comparability, in the expanded dataset we computed participants’ verbal WM span based on their performance on the digit backward span tasks. The VIF values of the predictors included in Model 2 ranged from 1.06 to 3.05. Hence, there was no harmful multicollinearity and we did not have to remove any of the variables above. It should also be noted that the vast majority of the neurotypical individuals who participated in Fyndanis et al.’s (2018) study were administered both lists of the sentence completion task.

**Figure 1:**
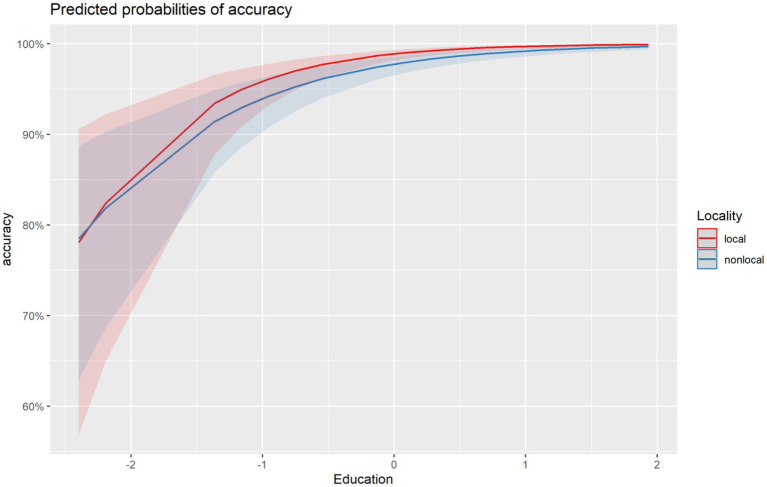
Interaction between (years of formal) Education and Locality (based on Model 2).

As shown in Table 5, the results of Model 2 replicated the main finding of Model 1, which was fitted to the original dataset of the current study. That is, the only significant interaction found was that between Locality and Education, with the latter affecting accuracy performance on verb-related morphosyntactic production more in local than in nonlocal configurations (Figure 1).^6^

To investigate whether the interaction between Locality and Education was modulated by the morphosyntactic categories considered here, we also fitted [to both the original dataset of the current study (*n* = 80) and to the expanded dataset (*n* = 140)] four models that included a three-way interaction between Locality, Education, and Morphosyntactic Condition as a fixed term, and differed in the random structure: the first model included subjects and items as random intercepts only; the second model included subjects and items as random intercepts, and Locality and Morphosyntactic Condition as by-subject random slopes; the third model included subjects and items as random intercepts, and Locality as by-subject random slope; and the fourth model included subjects and items as random intercepts, and Morphosyntactic Condition as by-subject random slope. The results of the best-fitting model (based on AIC; Model 3, Table 6) show that, in either dataset, there was no significant three-way interaction between Locality, Education, and Morphosyntactic Condition. Therefore, the significant interaction between Locality and Education was not modulated by the three morphosyntactic categories under investigation.

**Table 6:** Generalized linear mixed-effects Model 3 on accuracy.

	Estimate	Std. error	*z* value	*Pr* (> | *z* |)
*Model 3 (fitted to original dataset)*				
Intercept (Locality = Local; Morphosynt. Cond = Agreement)	6.073	0.503	12.073	<0.001^*^
Locality = Nonlocal	−1.365	0.525	−2.603	<0.01^*^
Education (years)	0.555	0.380	1.462	0.144
Morphosynt. Cond. = Aspect	−4.117	0.512	−8.037	<0.001^*^
Morphosynt. Cond. = Tense	−1.377	0.572	−2.409	0.016^*^
Locality = Nonlocal: Education	0.010	0.410	0.025	0.980
Locality = Nonlocal: Morphosynt. Cond. = Aspect	0.836	0.551	1.518	0.129
Locality = Nonlocal: Morphosynt. Cond. = Tense	1.059	0.610	1.735	0.083
Education: Morphosynt. Cond. = Aspect	0.581	0.387	1.502	0.133
Education: Morphosynt. Cond. = Tense	0.807	0.411	1.965	0.049^*^
Locality = Nonlocal: Education: Morphosynt. Cond. = Aspect	−0.302	0.419	−0.721	0.471
Locality = Nonlocal: Education: Morphosynt. Cond. = Tense	−0.683	0.468	−1.459	0.145
*Model 3 (fitted to expanded dataset)*				
Intercept (Locality = Nonlocal; Morphosynt. Cond = Agreement)	5.883	0.361	16.301	<0.001^*^
Locality = Nonlocal	−0.622	0.376	−1.652	0.098
Education (years)	0.987	0.256	3.852	<0.001^*^
Morphosynt. Cond. = Aspect	−3.505	0.365	−9.592	<0.001^*^
Morphosynt. Cond. = Tense	−0.360	0.466	−0.773	0.440
Locality = Nonlocal: Education	−0.222	0.275	−0.808	0.419
Locality = Nonlocal: Morphosynt. Cond. = Aspect	−0.016	0.404	−0.039	0.969
Locality = Nonlocal: Morphosynt. Cond. = Tense	0.084	0.448	0.187	0.852
Education: Morphosynt. Cond. = Aspect	0.505	0.247	2.048	0.041^*^
Education: Morphosynt. Cond. = Tense	0.979	0.313	3.130	0.002^*^
Locality = Nonlocal: Education: Morphosynt. Cond. = Aspect	−0.040	0.279	−0.143	0.887
Locality = Nonlocal: Education: Morphosynt. Cond. = Tense	0.042	0.317	0.134	0.894

Model 3 included the three-way interaction between Locality, Education, and Morphosyntactic Condition, Subjects and Items as random intercepts, and Locality and Morphosyntactic Condition as by-subject random slopes. The symbol * indicates significant effects.

**Figure 2:**
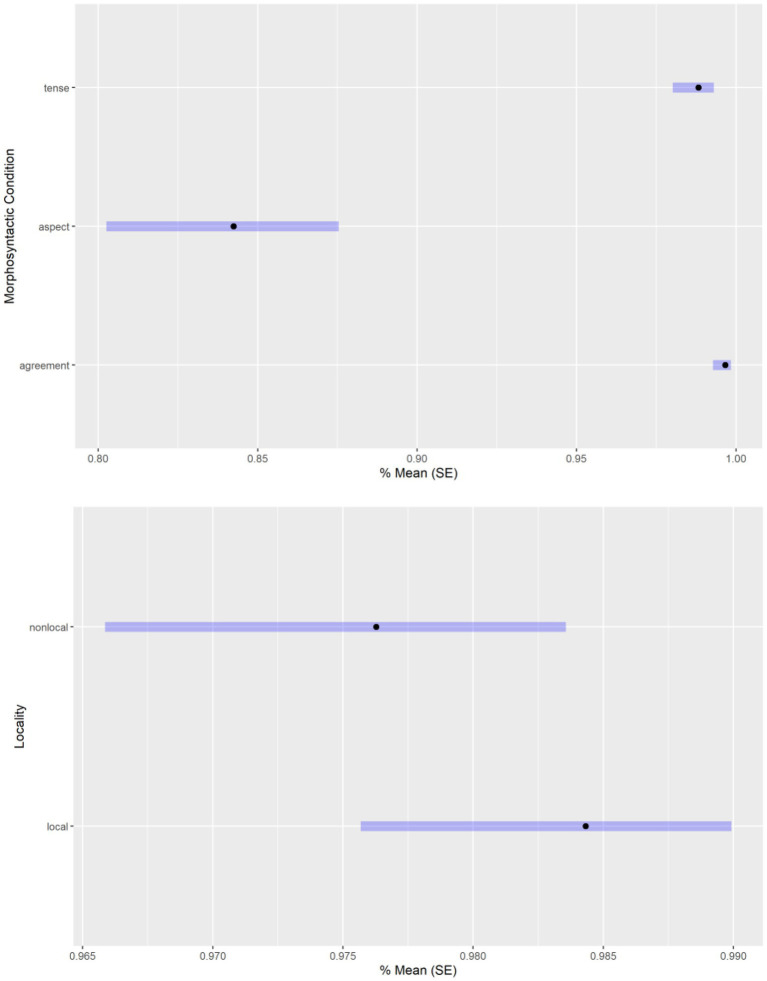
Top: Participants’ estimated percent correct performance (% mean) and SE on the production of grammatical Aspect, subject–verb Agreement, and Time Reference/Tense (with local and nonlocal configurations collapsed) based on Model 5. Bottom: Participants’ estimated correct % mean performance and SE on verb-related morphosyntactic production in local and nonlocal configurations (with Aspect, Agreement and Tense collapsed) based on Model 5.

To investigate the relationship between verb-related morphosyntactic production and the cognitive capacities considered here, we first fitted a model including Morphosyntactic Condition (three levels: Aspect, Agreement, Tense), Verbal WM capacity (continuous variable), Nonverbal WM capacity (continuous variable), Verbal STM capacity (continuous variable), Nonverbal STM capacity (continuous variable), SOP (continuous variable), (years of formal) Education/Long-Term WM for language (continuous variable), and Locality as fixed effects, as well as subjects and items as random intercepts. Subsequently, we fitted the same model as above with the addition of Morphosyntactic Condition and/or Locality as by-subject random slope(s). We selected the best-fitting model based on AIC (Model 4; see Table 7), and then calculated the VIF for all the predictors included in this model. The VIF values ranged from 1.03 to 1.81, which are considered acceptable (e.g., Akinwande et al., 2015); therefore, we did not have to remove any of the variables above. We then fitted additional models whose fixed effects were only the variables that showed significant main effects in Model 4. These models only differed in the random structure; they all included subjects and items as random intercepts, but they differed as for the presence of one vs. two random slopes [i.e., Morphosyntactic Condition and/or Locality as by-subject random slope(s)]. Again, we selected the best-fitting model based on AIC (Model 5; Table 7). The results of both Model 4 and Model 5 (Table 7) show that, in addition to the significant main effect of Morphosyntactic Condition, there were significant main effects of Verbal WM capacity, Verbal STM capacity, Education, and Locality. Overall, the greater the participants’ verbal STM capacity and verbal WM capacity, and the higher their educational level, the better their performance on verb-related morphosyntactic production. In Model 5, we also calculated the estimated marginal means (EMMs) using the emmeans R package (Russell, 2020) to provide *post hoc* comparisons between the three levels of Morphosyntactic Condition, that is, grammatical Aspect, Time Reference/Tense, and subject–verb Agreement (with results averaged over the two levels of Locality), and between the two levels of Locality, that is, Local and Nonlocal (with results averaged over the three levels of Morphosyntactic Condition). The results of these comparisons revealed that all differences were significant (see Table 8; Figure 2): participants fare better on subject–verb Agreement than on Time Reference/Tense and grammatical Aspect, and better on Time Reference/Tense than on grammatical Aspect; moreover, participants fare better in local configurations than in nonlocal configurations. Subsequently, we fitted two models that included the variables showing significant main effects in Models 4–5, namely Morphosyntactic Condition, Verbal WM Capacity, Verbal STM Capacity, (years of formal) Education and Locality, as well as interaction terms (Morphosyntactic Condition x each of the other fixed effects above). Again, these models only differed in their random structure, and we selected the best-fitting model based on AIC (Model 6; Table 9).

**Table 7:** Generalized linear mixed-effects Model 4 and Model 5 on accuracy.

	Estimate	Std. error	*z* value	*Pr* (> | *z* |)
*Model 4*				
Intercept (Morphosynt. Cond. = Agreement; Locality = Local)	5.904	0.412	14.318	<0.001^*^
Morphosynt. Cond. = Aspect	−4.008	0.394	−10.184	<0.001^*^
Morphosynt. Cond. = Tense	−1.241	0.427	−2.904	<0.01^*^
Verbal WM capacity	0.375	0.134	2.806	<0.01^*^
Verbal STM capacity	0.301	0.112	2.684	<0.01^*^
Nonverbal WM capacity	−0.055	0.118	−0.467	0.640
Nonverbal STM capacity	−0.072	0.114	−0.629	0.529
SOP	−0.022	0.140	−0.156	0.876
Education (years)	0.531	0.123	4.322	<0.001^*^
Locality = Nonlocal	−0.435	0.194	−2.245	0.025^*^
*Model 5*				
Intercept (Morphosynt. Cond. = Agreement; Locality = Local)	5.890	0.412	14.301	<0.001^*^
Morphosynt. Cond. = Aspect	−4.003	0.395	−10.147	<0.001^*^
Morphosynt. Cond. = Tense	−1.249	0.429	−2.914	<0.01^*^
Verbal WM capacity	0.349	0.128	2.726	<0.01^*^
Verbal STM capacity	0.265	0.107	2.479	0.013^*^
Education (years)	0.537	0.116	4.624	<0.001^*^
Locality = Nonlocal	−0.423	0.193	−2.189	0.029^*^

Model 4 included the additive effect of Morphosyntactic Condition, Verbal WM Capacity, Nonverbal WM Capacity, Verbal STM Capacity, Nonverbal STM Capacity, SOP, (years of formal) Education, and Locality, Subjects and Items as random intercepts, and Morphosyntactic Condition and Locality as by-subject random slopes. Model 5 included the additive effect of Morphosyntactic Condition, Verbal WM Capacity, Verbal STM Capacity, (years of formal) Education and Locality, Subjects and Items as random intercepts, and Morphosyntactic Condition and Locality as by-subject random slopes. The symbol * indicates significant effects.

**Table 8:** Between-morphosyntactic category and between-locality comparisons.

Contrast	Odds.Ratio	*SE*	*z* ratio	*p*
Agr/Asp	54.753	21.599	10.147	<0.001^*^
Agr/T	3.487	1.495	2.914	0.01^*^
Asp/T	0.064	0.017	−10.236	<0.001^*^
Local/Nonlocal	1.530	0.294	2.189	0.029^*^

Agr, subject–verb Agreement; Asp, grammatical Aspect; and T, Tense/Time Reference. The symbol * indicates significant effects.

**Table 9:** Generalized linear mixed-effects Model 6 and Model 7 on accuracy.

	Estimate	Std. error	*z* value	*Pr* (> | *z* |)
*Model 6*				
Intercept (Morphosynt. Cond. = Agreement; Locality = Local)	6.161	0.472	13.067	<0.001^*^
Morphosynt. Cond. = Aspect	−4.248	0.476	−8.931	<0.001^*^
Morphosynt. Cond. = Tense	−1.701	0.516	−3.293	<0.001^*^
Verbal WM capacity	−0.460	0.300	−1.532	0.125
Verbal STM capacity	0.208	0.278	0.746	0.456
Education (years)	0.732	0.292	2.509	0.012^*^
Locality = Nonlocal	−1.469	0.462	−3.178	0.001^*^
Morphosynt. Cond. = Aspect: Verbal WM capacity	0.916	0.316	2.902	0.004^*^
Morphosynt. Cond. = Tense: Verbal WM capacity	0.669	0.346	1.935	0.053
Morphosynt. Cond. = Aspect: Verbal STM capacity	0.063	0.291	0.215	0.830
Morphosynt. Cond. = Tense: Verbal STM capacity	0.380	0.313	1.216	0.224
Morphosynt. Cond. = Aspect: Education	−0.210	0.304	−0.690	0.490
Morphosynt. Cond. = Tense: Education	−0.158	0.322	−0.491	0.624
Morphosynt. Cond. = Aspect: Locality = Nonlocal	1.021	0.485	2.104	0.035^*^
Morphosynt. Cond. = Tense: Locality = Nonlocal	1.570	0.514	3.053	0.002^*^
*Model 7*				
Intercept (Morphosynt. Cond. = Agreement; Locality = Local)	6.153	0.462	13.320	<0.001^*^
Morphosynt. Cond. = Aspect	−4.236	0.467	−9.080	<0.001^*^
Morphosynt. Cond. = Tense	−1.746	0.498	−3.508	<0.001^*^
Verbal WM capacity	−0.390	0.233	−1.675	0.094
Verbal STM capacity	0.267	0.107	2.499	0.012^*^
Education (years)	0.548	0.117	4.686	<0.001^*^
Locality = Nonlocal	−1.472	0.463	−3.180	0.001^*^
Morphosynt. Cond. = Aspect: Verbal WM capacity	0.837	0.232	3.607	<0.001^*^
Morphosynt. Cond. = Tense: Verbal WM capacity	0.744	0.253	2.940	<0.01^*^
Morphosynt. Cond. = Aspect: Locality = Nonlocal	1.026	0.486	2.110	0.035^*^
Morphosynt. Cond. = Tense: Locality = Nonlocal	1.570	0.514	3.055	0.002^*^
Intercept (Morphosynt. Cond. = Aspect; Locality = Local)	1.916	0.198	9.696	<0.001^*^
Morphosynt. Cond. = Agreement	4.236	0.467	9.074	<0.001^*^
Morphosynt. Cond. = Tense	2.490	0.321	7.760	<0.001^*^
Verbal WM capacity	0.447	0.126	3.532	<0.001^*^
Verbal STM capacity	0.267	0.107	2.499	0.012^*^
Education (years)	0.548	0.117	4.686	<0.001^*^
Locality = Nonlocal	−0.446	0.223	−1.998	0.046^*^
Morphosynt. Cond. = Agreement: Verbal WM capacity	−0.837	0.232	−3.606	<0.001^*^
Morphosynt. Cond. = Tense: Verbal WM capacity	−0.093	0.207	−0.447	0.655
Morphosynt. Cond. = Agreement: Locality = Nonlocal	−1.026	0.486	−2.109	0.035^*^
Morphosynt. Cond. = Tense: Locality = Nonlocal	0.544	0.350	1.553	0.120

Model 6 included the additive effect of Morphosyntactic Condition, Verbal WM Capacity, Verbal STM Capacity, (years of formal) Education and Locality, the interactions between (1) Morphosyntactic Condition and Verbal WM Capacity, (2) Morphosyntactic Condition and Verbal STM Capacity, (3) Morphosyntactic Condition and Education, and (4) Morphosyntactic Condition and Locality, Subjects and Items as random intercepts, and Morphosyntactic Condition and Locality as by-subject random slopes. Model 7 included the additive effect of Morphosyntactic Condition, Verbal WM Capacity, Verbal STM Capacity, (years of formal) Education, and Locality, the interaction between Morphosyntactic Condition and Verbal WM Capacity, the interaction between Morphosyntactic Condition and Locality, Subjects and Items as random intercepts, and Morphosyntactic Condition and Locality as by-subject random slopes. The symbol * indicates significant effects.

Finally, we fitted models that included the significant interaction terms and the variables showing significant main effects in Model 6, that is, Morphosyntactic Condition, Verbal WM Capacity, Verbal STM Capacity, (years of formal) Education, and Locality, as well as the interaction between Morphosyntactic Condition and Verbal WM Capacity, and the interaction between Morphosyntactic Condition and Locality. Again, these models only differed in the random structure. The results of the best-fitting model (Model 7) are presented in Table 9 (note that, in Table 9, we present the results of Model 7 using two different intercepts to find out not only if verbal WM affects Aspect and Tense/Time Reference significantly more than Agreement, but also if it affects Aspect significantly more than Tense/Time Reference).

**Figure 3:**
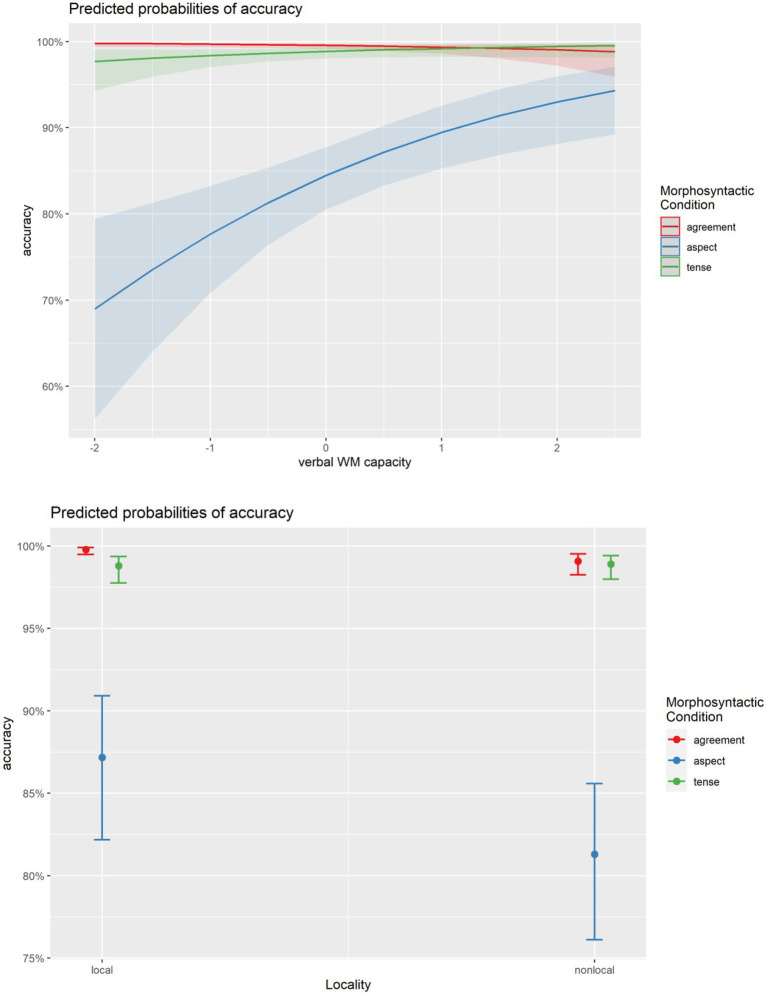
Top: Interaction between morphosyntactic categories and verbal WM capacity based on Μodel 7. Bottom: Interaction between morphosyntactic categories and locality (based on Model 7).

The results show that the production of the three morphosyntactic categories significantly interacted with verbal WM capacity, but not with verbal STM capacity or education (Model 6; Table 9). In particular, verbal WM capacity affected Aspect and Time Reference significantly more than Agreement [Model 7; Table 9; see also Figure 3 (top)]. There was no differential effect of verbal WM capacity on Aspect and Time Reference (Model 7; Table 9). Lastly, the three morphosyntactic categories significantly interacted with Locality (see Model 7, Table 9), with the latter affecting Aspect and Agreement, but not Tense/Time Reference (Figure 3, bottom). That Locality affected Aspect and Agreement (with nonlocal Aspect/Agreement eliciting more errors than local Aspect/Agreement) but not Tense/Time Reference can be seen not only in Figure 3 (bottom), but also in the output of *post hoc* comparisons (based on EMMs, Model 7) between local and local Aspect, local and nonlocal Agreement, and local and nonlocal Tense/Time Reference (Table 10).

**Table 10:** Comparisons between local and nonlocal subject–verb Agreement, local and nonlocal grammatical Aspect, and local and nonlocal Tense/Time Reference.

Contrast	Odds.Ratio	*SE*	*z* ratio	*p*
Local Agreement/Nonlocal Agreement	4.358	2.017	3.180	<0.01^*^
Local Aspect/Nonlocal Aspect	1.563	0.349	1.998	0.046^*^
Local Tense/Nonlocal Tense	0.907	0.286	−0.310	0.756

The symbol * indicates significant effects.

Finally, we computed the Kuder–Richardson Formula 20 (KR-20) for the grammatical Aspect, Time Reference, and subject–verb Agreement conditions based on the expanded dataset, and specifically based on the data of the participants who completed both lists of the sentence completion task (*n* = 40). Results showed that there was excellent internal consistency for the Aspect and Time Reference conditions (for both, KR-20 = 0.98) and very good internal consistency for the Agreement condition (KR-20 = 0.83).

### Error Analysis

Overall, 754 errors occurred. The vast majority of these errors (735; i.e., 97.5%) were repetition-type errors. That is, the participant produced a verb form that encoded the same value(s) of the morphosyntactic feature of interest (i.e., grammatical aspect, time reference, person or number) that was/were encoded on the verb form included in the source sentence. For example, in the grammatical Aspect condition, repetition-type errors were verb productions that encoded the aspectual value—perfective or imperfective—appearing in the source sentence. In the Time Reference condition, repetition-type errors occurred when the participant produced a verb form that referred to the same time frame—past or future—as that of the verb form that appeared in the source sentence. Lastly, in the subject–verb Agreement condition, repetition-type errors occurred when the participant produced a verb form that encoded the same number (singular or plural) or person (first, second or third) as those encoded on the grammatical subject of the source sentence.

Of the total number of the 754 errors, 600 occurred in the Aspect condition, 120 in the Time Reference condition, and 34 in the Agreement condition. There were 598, 110, and 33 repetition-type errors in the Aspect, Time Reference, and Agreement conditions, respectively. Of the 600 aspect errors, 350 occurred in the imperfective aspect condition and 250 occurred in the perfective aspect condition. Of the 120 time reference errors, 70 occurred in the past reference subcondition and 50 in the future reference subcondition. Lastly, of the 34 agreement errors, 27 occurred in the number agreement subcondition and seven in the person agreement subcondition.

## Discussion

Following up on Fyndanis et al. (2018), this study focused on neurologically healthy Greek-speaking middle-aged and older participants and investigated the relationship between verb-related morphosyntactic production and verbal WM, nonverbal/visuospatial WM, verbal STM, nonverbal/visuospatial STM, SOP, (years of formal) education, and locality, as well as the relationship between locality and the memory systems above (including “long-term WM for language” (Caplan and Waters, 2013), for which education was used as a proxy). We found significant main effects of verbal WM capacity, verbal STM capacity, education, and locality: the greater the verbal WM/STM capacity and the higher the educational level, the better the verb-related morphosyntactic production; verb-related morphosyntactic production was better in local than in nonlocal configurations. Moreover, there were significant interactions between verbal WM capacity and verb-related morphosyntactic production, between locality and education, and between locality and the three morphosyntactic categories under consideration. More specifically, verbal WM capacity affected grammatical Aspect and Time Reference/Tense more than subject–verb Agreement; education affected participants’ performance more in local than in nonlocal configurations; and locality affected Aspect and Agreement (with nonlocal Aspect/Agreement eliciting more errors than local Aspect/Agreement) but not Time Reference/Tense.

### Discussion of Locality Effects

The present findings about locality partly contradict Fyndanis et al.’s (2018) study, in which there was no significant main effect of locality. This discrepancy might be due to different analyses of the data performed in the two studies. Unlike in the current study, Fyndanis et al. (2018) had to address convergence issues and decided to remove items from the random structure and to average accuracy on all items per locality and morphosyntactic conditions across subjects. They transformed thus the dichotomous dependent variable into a continuous one, resulting in loss of information on item variability (Baayen, 2008). It should be noted that, unlike in the current study, the relationship between locality and education was not investigated in Fyndanis et al.’s (2018) study. Furthermore, Fyndanis et al. only checked whether locality interacted with verbal WM capacity and morphosyntactic condition including a three-way interaction. This interaction was not significant, but this result cannot be compared with any of the interactions including locality reported in the current study.

As far as the significant main effect of locality is concerned, overall, nonlocal configurations/dependencies elicited more morphosyntactic errors than local configurations/dependencies. Since the vast majority of errors occurred in the grammatical Aspect condition, the locality effect was primarily driven by this morphosyntactic category. This result could be explained by assuming that, when the critical cue (e.g., aspectual adverbial in the Aspect condition) is not adjacent to the target verb form, more demands are posed on the STM/WM system as the aspectual feature that is extracted from the critical cue should be maintained for a longer time as compared to local configurations, in which the critical cue is adjacent to the verb. Nevertheless, the absence of interaction between locality and STM/WM does not support the view that the effect of locality is due to STM/WM. An alternative explanation could be that, at least in Greek, aspectual adverbials are more frequently placed next to the verb than in a more distant position, and this frequency difference between local and nonlocal configurations for grammatical Aspect has trained—through lifelong exposure to language and through language production—the “language processor” of Greek-speaking individuals to be more efficient when handling grammatical Aspect in local configurations. Assuming that education is related to experienced-based aspects of language learning (including statistical learning), this explanation is consistent with the significant interaction between education and locality, with the former affecting morphosyntactic production more in local than in nonlocal configurations.

This possibility leads to discussion of the interaction of education and locality. It should be noted that the interpretation of the role of education depends on the view of WM one adopts. Here, we adopted the multicomponent view of WM (e.g., Baddeley, 1986; Martin and Romani, 1994; Martin et al., 1994, 1999) and the notion that language is supported not only by a controlled STM/WM system that is distinct from long-term memory, but also by a procedural memory system that is specialized for language, which might be “long-term WM for language” (Caplan and Waters, 2013). In this approach to memory, education might be related to the efficiency of long-term WM for language, and the significant interaction between locality and education could be accounted for by assuming that long-term WM for language—which is shaped by experience with language and captures frequency patterns in language—interacts with “local Aspect” and “nonlocal Aspect” because the former is more frequent in Greek than the latter. That is, on the assumption that long-term WM for language better supports aspects of language performance in the most “favorable” linguistic environments (i.e., in the environments where the “language processor” has been exposed and trained the most) rather than in less favorable environments, and if aspectual adverbials are usually placed next to the verb in Greek, it is reasonable that long-term WM for language better supports production of grammatical Aspect in local (rather than in nonlocal) configurations.

A slightly different interpretation of the role of education emerges from the experience-based approach to verbal WM, which posits that verbal WM is a skill that emerges from “actions” of the language systems and varies with experience (e.g., MacDonald and Christiansen, 2002; MacDonald, 2016; Schwering and MacDonald, 2020), and interprets performance on verbal STM/WM tasks to reflect quality and quantity of language skill and experience (Schwering and MacDonald, 2020). On this approach, one could assume that both verbal WM capacity and education are measures of language skill and experience. It might be that education is related to the aspect of language experience that pertains to statistical learning and captures information about the relative frequency with which aspectual adverbials appear adjacent vs. nonadjacent to the verb. If aspectual adverbials are usually placed next to the verb in Greek, it is the experience-based language skill (captured by education) that favors local configurations.

One could challenge the above interpretation assuming that what matters may be the proportion of times that one encounters local vs. nonlocal configurations (for a given morphosyntactic category), not the raw number of times. Academic writing presumably includes a higher proportion of nonlocal configurations than conversational speech, and thus, the most educated individuals should be exposed to a greater proportion of nonlocal configurations than the less educated individuals. We assume, however, that even if the most educated individuals are exposed to a higher proportion of nonlocal conditions compared to the less educated individuals, still both the more and the less educated individuals are exposed to more local than nonlocal configurations for grammatical aspect. We argue, therefore, that the content of the interaction between education and locality can be accounted for by assuming that more years of education lead to more exposure to language and “better statistical learning” of the most frequent configurations in which grammatical aspect occurs, which are presumably local configurations. The assumption that local configurations outnumber nonlocal configurations for grammatical aspect in both academic writing and conversational speech has to be tested in future research.

The significant interaction between locality and the three morphosyntactic categories, which shows that locality affected grammatical Aspect and subject–verb Agreement but not Time Reference/Tense, could not reflect a ceiling effect as participants fared worse on Time Reference/Tense than on Agreement (120 and 34 errors, respectively). It might be that, in Greek, local configurations for Aspect and Agreement are more frequent than nonlocal configurations for these two morphosyntactic categories, whereas local and nonlocal configurations for Time Reference/Tense are equally frequent. Thus, this interaction may reflect the statistical distribution of local vs. nonlocal Aspect, Agreement, and Time Reference/Tense in Greek.

### Discussion of Verbal STM/WM and Education Effects

As mentioned above, the significant main effects of verbal WM, verbal STM, and education showed that the greater the participants’ verbal STM/WM capacity, and the higher their educational level, the better their performance on verb-related morphosyntactic production. However, of these three predictor variables, only verbal WM capacity significantly interacted with the language task performance as it affected grammatical Aspect and Time Reference/Tense more than subject–verb Agreement. This interaction results from participants’ ceiling performance on Agreement. If the sentences included in the agreement condition had favored agreement attraction errors, presumably this condition would have also drawn on WM (in line with the findings reported in Hartsuiker and Barkhuysen, 2006, and Slevc and Martin, 2016) and would have elicited more errors, possibly resulting in a no significant interaction between verbal WM capacity and the three morphosyntactic categories. However, in the current study, the agreement condition did not favor attraction errors, as in the nonlocal dependencies included in this condition, “none of the intervening phrases was a postmodifier of the head noun phrase (the subject)” and “all subjects were animate, and all intervening noun phrases (which were part of the aspectual adverbials) were inanimate” (Fyndanis et al., 2018, p. 1175). As mentioned in Fyndanis et al. (2018), prepositional phrases modifying or complementing the subject noun phrase are more “semantically integrated” with it than prepositional phrases not modifying it (Solomon and Pearlmutter, 2004), and this factor favors agreement attraction errors. Moreover, animacy may play a role in determining subjecthood (Bock et al., 1992); in agreement, the number and person values of the animate subject might be more resistant to cue-based retrieval interference compared to configurations such as *The key to the cabinets*…, in which both nouns are inanimate.

Moreover, participants fared significantly better on Agreement than on Time Reference and Aspect, and significantly better on Time Reference than on Aspect. These results largely replicate Fyndanis et al.’s (2018) results as the same pattern of performance and a similar interaction emerged: both the present study and Fyndanis et al.’s (2018) study found that verbal WM affects grammatical Aspect and Time Reference more than subject–verb Agreement. Nevertheless, unlike Fyndanis et al. (2018), the present results do not show a differential effect of verbal WM on Aspect and Time Reference. The partial discrepancy in results between the two studies might be due to different approaches to data analysis followed in the two studies (for details, see *Discussion of Locality Effects*).

That verbal WM and verbal STM (but not nonverbal STM/WM) are involved in verb-related morphosyntactic production suggests that production of verb-related morphosyntactic categories is predominantly supported by domain-specific (and not domain-general) memory resources. This is so because verbal and nonverbal STM systems predominantly rely on domain-specific resources (e.g., Kane et al., 2004; Hanley and Young, 2019; Logie, 2019), and verbal WM relies on domain-general resources to a lesser extent than nonverbal WM (e.g., Vergauwe et al., 2010).

The significant main effects of verbal WM and verbal STM suggest that both the processing and storage components of WM are relevant to verb-related morphosyntactic production. This is at odds with Fyndanis et al. (2018, pp. 1183–1184) who argued that “the aspect of WM that is relevant to the processing of tense and aspect is not maintenance of item or item-and-order information,” but “its role in processing information and/or, in this task, suppressing responses based on previously presented information.”

That verbal WM (but not verbal STM) significantly interacted with the production of grammatical Aspect, Time Reference/Tense, and subject–verb Agreement, and the fact that verbal WM partly shaped the pattern of performance reported here suggest that the production of grammatical Aspect and Time Reference/Tense is *computationally* more demanding than the production of subject–verb Agreement. This may be due to qualitative differences in these aspects of sentence form and meaning. That verbal WM affected Aspect and Time Reference/Tense more than subject–verb Agreement is also consistent with IFIH (Fyndanis et al., 2012), which posits that categories bearing interpretable features (e.g., Aspect, Tense) are computationally more costly than categories bearing uninterpretable features (e.g., subject–verb Agreement; see also Varlokosta et al., 2006).

That education predicted participants’ performance on a “laboratory language task” tapping into verb-related morphosyntactic production is consistent with the view that the higher one’s education, the more increased their experience in formal testing situations (Ostrosky-Solis et al., 1998). Furthermore, assuming that education level determines the degree of linguistic experience and, therefore, also the strength of the connections in the linguistic network hosted in long-term memory, this demographic factor could be argued—as mentioned above—to be a proxy of another memory system, i.e., long-term WM for language (e.g., Caplan and Waters, 2013), which appears to play a role in verb-related morphosyntactic production.

Turning to the absence of a significant main effect of SOP, it should be noted that, as expected, in the present study SOP was positively and significantly correlated with both verbal WM (*r* = 0.53, *p* < 0.001) and nonverbal WM (*r* = 0.51, *p* < 0.001). This is consistent with earlier findings showing that SOP is closely related to WM (e.g., Salthouse, 1992; Fry and Hale, 1996, 2000). Since the role of SOP in verb-related morphosyntactic production was investigated together with the role of WM, the absence of a significant main effect of SOP might be accounted for by assuming that the measure of morphosyntactic performance used here (i.e., accuracy in an off-line/untimed task) is not sensitive to the component of SOP that does not overlap with WM. Alternatively, it may be that the measure of SOP used here is weighted toward perceptual tasks, and speech production is weighted toward motor tasks.

Finally, a different interpretation of these results could be offered by the experience-based/emergent view of verbal STM/WM (e.g., MacDonald and Christiansen, 2002; MacDonald, 2016; Schwering and MacDonald, 2020). First, on the assumption that participants’ performance on verbal STM/WM tasks reflect quality and quantity of language skill and experience (Schwering and MacDonald, 2020), and given that the grammatical Aspect and Time Reference/Tense conditions elicited significantly more errors than the subject–verb Agreement condition, one could argue that Aspect and Time Reference are more sensitive indices of quantity and quality of language skill and experience than Agreement. The significant main effect of education on verb-related morphosyntactic production could be accounted for by assuming—as mentioned above—that education is another proxy for language skill. This is consistent with the strong correlation between verbal WM capacity and education in the datasets analyzed here (*r* = 0.59 in both the original and expanded datasets). The fact that only verbal WM (and not verbal STM or education) significantly interacted with the production of grammatical Aspect, Time Reference, and Agreement could be interpreted as suggesting that verbal WM tasks are better measures of language skill than verbal STM tasks or years of formal education. Therefore, the better the participants’ language skill, the better their performance on verbal WM tasks and the better their accuracy performance on demanding morphosyntactic categories such as grammatical Aspect and Time Reference/Tense. In contrast, subject–verb Agreement is an undemanding morphosyntactic category that elicits ceiling performance.

## Conclusion

To sum up, the current study investigated the role of verbal and nonverbal STM and WM, SOP, education, and locality in the production of grammatical Aspect, Time Reference/Tense, and subject–verb Agreement in Greek. There were significant main effects of verbal WM, verbal STM, and education: the greater the participants’ verbal STM/WM capacity, and the higher their educational level, the better their accuracy performance on verb-related morphosyntactic production. Also, there was a significant main effect of locality, with nonlocal configurations eliciting more morphosyntactic errors than local configurations. Moreover, locality significantly interacted with education (with the latter affecting morphosyntactic production more in local than in nonlocal configurations) and the three morphosyntactic categories (with locality affecting Aspect and Agreement but not Time Reference/Tense). The interaction between locality and education could be accounted for by assuming that education is an index of a procedural memory system which is sensitive to frequency patterns in language and better supports verb-related morphosyntactic production in more frequent than in less frequent configurations. Similarly, the interaction between locality and the three morphosyntactic categories might reflect the statistical distribution of local vs. nonlocal Aspect, Agreement, and Time Reference/Tense in Greek. Lastly, a significant interaction between verbal WM and the three morphosyntactic categories emerged, with verbal WM affecting Aspect and Time Reference/Tense more than Agreement. That verbal STM/WM (but not nonverbal STM/WM) supported the production of the morphosyntactic categories above suggests that verb-related morphosyntactic production predominantly recruits domain-specific (and not domain-general) memory resources. The significant main effects of both verbal WM and verbal STM suggest that both the processing and storage components of WM are relevant to verb-related morphosyntactic production. That verbal WM (but not verbal STM) interacted with the production of Aspect, Time Reference/Tense, and Agreement suggests that Aspect and Time Reference/Tense are computationally more demanding than Agreement.

## Data Availability Statement

The datasets supporting the conclusions of this article are available on request to the corresponding author.

## Ethics Statement

This study was approved by the Research Ethics Committee of the School of Psychology (Faculty of Philosophy) at the Aristotle University of Thessaloniki. The patients/participants provided their written informed consent to participate in this study.

## Author Contributions

VF, EM, and DC contributed to the conception and design of the study. EC and ID contributed to participant recruitment, data collection, and data transcription. SM and VF analyzed the data. VF, EM, and SM wrote the first draft of the manuscript. All authors contributed to the revision of the first draft and approved the submitted version.

## Funding

This work was partly supported by the Research Council of Norway through its Centers of Excellence funding scheme (project number 223265) and FRIPRO funding scheme (project number 287745).

## Conflict of Interest

The authors declare that the research was conducted in the absence of any commercial or financial relationships that could be construed as a potential conflict of interest.

## Publisher’s Note

All claims expressed in this article are solely those of the authors and do not necessarily represent those of their affiliated organizations, or those of the publisher, the editors and the reviewers. Any product that may be evaluated in this article, or claim that may be made by its manufacturer, is not guaranteed or endorsed by the publisher.

## Figures and Tables

**Figure:**
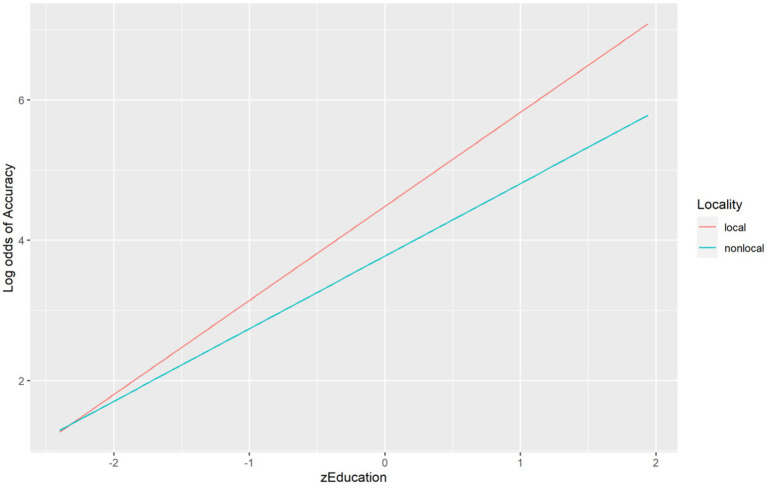
Log odds ratio of accuracy with respect to the interaction between Education and Locality.

## Footnotes

^1^According to the Minimalist Program, the number and person features encoded on the subject are interpretable. However, these features encoded on the verb are uninterpretable. It should be noted, however, that there are opposing theoretical views on the (un)interpretability of person and number features encoded on the verb when it comes to pro-drop/null-subject languages such as Greek (for details, see Fyndanis et al., 2012, and reference therein). The theoretical distinction between interpretable and uninterpretable features has been influential not only in aphasia research (see Garraffa and Fyndanis, 2020), but also in second language acquisition research (see, for example, Tsimpli and Dimitrakopoulou, 2007).

^2^Dosi, I. (2016). The Verbal Aspect in Bilingual Children: The Effect of Linguistic, Cognitive and Environmental Factors. Unpublished PhD Thesis. Aristotle University of Thessaloniki.

^3^For example, the sentence *The key to the cabinets is/*are on the table* involves cue-based retrieval interference, as the degree to which *key* and *cabinets* match cues derived from the verb may result in subject-verb agreement errors (for cue-based retrieval interference in sentence comprehension, see Van Dyke and McElree, 2006).

^4^Fyndanis et al. (2018), for example, did not address this question as they assessed their participants with language and verbal WM tasks only.

^5^Of the WM models that adopt the multicomponent view, Martin and colleagues’ (Martin and Romani, 1994; Martin et al., 1994, 1999) model appears to be the most suitable one for storage of prephonological, lemma-level information. Such information is stored in the lexical-semantic buffer of STM/WM.

^6^The log odds ratio of accuracy with respect to the interaction between Education and Locality (based on Model 2) is depicted in the figure included in Appendix 1.
